# Candidate Gene Discovery in Hereditary Colorectal Cancer and Polyposis Syndromes–Considerations for Future Studies

**DOI:** 10.3390/ijms21228757

**Published:** 2020-11-19

**Authors:** Iris B. A. W. te Paske, Marjolijn J. L. Ligtenberg, Nicoline Hoogerbrugge, Richarda M. de Voer

**Affiliations:** 1 Department of Human Genetics, Radboud Institute for Molecular Life Sciences, Radboud University Medical Center, 6525 GA Nijmegen, The Netherlands; iris.tepaske@radboudumc.nl (I.B.A.W.t.P.); marjolijn.ligtenberg@radboudumc.nl (M.J.L.L.); nicoline.hoogerbrugge@radboudumc.nl (N.H.); 2 Department of Pathology, Radboud Institute for Molecular Life Sciences, Radboud University Medical Center, 6525 GA Nijmegen, The Netherlands

**Keywords:** colorectal tumors, genetic predisposition, missing heritability, molecular diagnosis, molecular biomarkers, rare variants

## Abstract

To discover novel high-penetrant risk loci for hereditary colorectal cancer (hCRC) and polyposis syndromes many whole-exome and whole-genome sequencing (WES/WGS) studies have been performed. Remarkably, these studies resulted in only a few novel high-penetrant risk genes. Given this observation, the possibility and strategy to identify high-penetrant risk genes for hCRC and polyposis needs reconsideration. Therefore, we reviewed the study design of WES/WGS-based hCRC and polyposis gene discovery studies (*n* = 37) and provide recommendations to optimize discovery and validation strategies. The group of genetically unresolved patients is phenotypically heterogeneous, and likely composed of distinct molecular subtypes. This knowledge advocates for the screening of a homogeneous, stringently preselected discovery cohort and obtaining multi-level evidence for variant pathogenicity. This evidence can be collected by characterizing the molecular landscape of tumors from individuals with the same affected gene or by functional validation in cell-based models. Together, the combined approach of a phenotype-driven, tumor-based candidate gene search might elucidate the potential contribution of novel genetic predispositions in genetically unresolved hCRC and polyposis.

## 1. Introduction

Colorectal cancer (CRC) is the third most commonly occurring cancer in men and the second most commonly observed cancer in women in Western society [1]. While the majority of individuals with CRC are not genetically predisposed, a predisposition to CRC may be considered when an individual is diagnosed with CRC at a young age (<50 years), when close relatives of the proband are affected with (colorectal) cancer, or when an individual has developed multiple adenomas in the colorectum. The presence of multiple adenomas (cumulative number of >10) at a young age (<60 years) is clinically diagnosed as polyposis.

The exact contribution of heritable factors to CRC and polyposis is still not fully understood. In the early 2000s Nordic twin- and family studies indicated that 12–35% of CRCs are potentially linked to heritable factors [2,3]. Later, estimates for heritability of CRC decreased to about 15% of all CRC cases [4,5]. At present, it is estimated that about 5–10% of all CRC and polyposis cases are explained by rare pathogenic variants in high-penetrant risk genes [2,6,7,8]. Next to identification of rare high-penetrant risk genes contributing to the heredity of CRC, it is estimated that common variants (minor allele frequency (MAF) in the general population >1%) may explain about ~12% of the relative risk for CRC [8,9,10,11].

The sum of these estimations covers largely the reported heritability in twin studies, but a substantial group of patients that fulfill the clinical criteria for hereditary CRC (hCRC) and/or polyposis are considered to remain without a genetic diagnosis [12,13,14,15]. It is important to resolve this missing heritability as a genetic diagnosis may favor a patient’s disease prognosis, surveillance and counseling, but may also have large implications for family members in disease prevention. It was anticipated that the rapid developments of next-generation sequencing (NGS) would aid in reducing the missing heritability for CRC and polyposis. However, despite numerous NGS studies, little additional high-penetrant novel risk genes for hCRC and polyposis have been found in the past decade. Over the years, various hypotheses for the missing heritability in hCRC and polyposis have been proposed and include the contribution of environmental factors and epigenetics, but also technical and methodological limitations of previous research [13,14]. To have a better insight into the reasons why NGS studies have not been able to resolve more high-penetrant risk genes for CRC and/or polyposis, we reviewed the strategies that have led to the identification of currently well-established hCRC and polyposis predisposing genes and all whole exome- and genome-based NGS studies aimed at the discovery of novel hCRC and polyposis risk genes. Each study was reviewed for its experimental setup to identify its methodological strengths and limitations of candidate gene discovery. Based on our findings and with the latest knowledge of hereditary cancer, we have formulated improvements for future research aimed at unraveling the genetic predisposition of unresolved hCRC and/or polyposis syndromes.

## 2. High-Penetrant Risk Genes Discovery in hCRC and Polyposis

**Figure 1:**
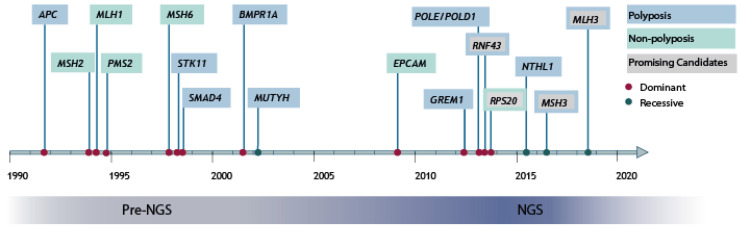
Genes associated with hereditary colorectal cancer (hCRC) and polyposis syndrome. Genes discovered over time for hCRC and polyposis. Established genes in full colors, promising risk genes with colored outline. NGS; Next-generation sequencing.

Most genes associated with hCRC and polyposis were discovered in the late 1900s, far before the NGS-era (Figure 1). In that time, linkage analysis of patients and families with early-onset CRC and/or polyp formation led to the discovery of Lynch syndrome, Familial Adenomatous Polyposis (FAP), and Hamartomatous Polyposis syndromes [16,17,18,19,20,21,22,23,24,25]. For these syndromes, genetic analysis of familial cases was supplemented with the analysis of cancer cell lines and (sporadic) tumors. Mismatch repair (MMR) associated genes *MSH2* and *MLH1* were discovered after the observation of linkage of microsatellite markers in multiple kindreds and instability of microsatellite repeats in tumors of affected relatives [20,21]. The observation that pathogenic variants in the MMR-genes *MSH2* and *MLH1* were the cause of familial CRC [22,23] led to the identification of *PMS2* as a cause for Lynch syndrome [24], followed by *MSH6* a couple of years later [25]. While the identification of these four genes explained the majority of individuals with MMR-deficient CRCs with a strong familial aggregation, several families remained without a genetics diagnosis, while their tumors revealed a deficiency of MSH2. It took almost another year before, in 2009, it was found that 3′ deletions of *EPCAM*, located upstream of *MSH2*, were involved in Lynch syndrome as well [26,27].

For discovery of FAP, linkage analysis of families in which polyposis affected multiple generations pointed toward the chromosome 5q21-22 region [17]. Additional analysis of tumors of sporadic CRC patients indicated that the 5q21 locus was frequently lost in tumor cells, pointing at a role for genes in this region in tumor development. Subsequently, germline pathogenic variants in *APC*, located in this 5q21 locus, were identified as the cause of the polyposis phenotype [19,28]. The observation of variable severity among FAP patients with regard to age-of-onset and polyp number led to the establishment of the ‘attenuated FAP’ (AFAP) phenotype. AFAP is linked to pathogenic variants outside the 3–15 exon regions of *APC* [18,29,30,31]. In the early 2000s, molecular phenotyping of tumors of three FAP-suspected siblings, but negative for germline pathogenic variants in *APC*, showed a shared increased rate of somatic C:G > A:T transversions in *APC* in their tumors. This observation led to the discovery of another adenomatous polyposis syndrome caused by biallelic pathogenic germline variants in the base-excision repair (BER) gene *MUTYH* [32], also known as *MUTYH*-associated polyposis (MAP). The discovery of more biallelic *MUTYH*-affected cases showed that MAP patients are equally associated with a classical FAP phenotype (>100 polyps) and an AFAP phenotype (<100 polyps) [33,34].

TGF-β signaling proteins *SMAD4* and *BMPR1A*, and AMPK-pathway activator *STK11*, were discovered by linkage and co-segregation studies in the late 1990s [35,36,37]. These genes are found to be associated with hamartomatous polyposis, characterized by juvenile polyps (for further reading; Zbuk and Eng 2007 [38]).

Subsequently, advances in sequencing techniques such as massive parallel sequencing allowed discovery approaches to change from targeted candidate gene sequencing to exome-wide and genome-wide sequencing of larger patient cohorts to identify disease-causing genes in a more hypothesis-free scenario. Over the past decade, multiple whole-exome and whole-genome sequencing-based studies have been performed (*n* = 37), all with the general aim to discover rare novel candidate risk genes for hCRC and polyposis (Table 1, Table S1). Thus far, these studies have resulted in over a hundred candidate genes for hCRC and/or polyposis, for which the majority have not been independently validated yet. Some genes are currently under debate as promising candidates for hCRC and polyposis, such as *RNF43*, *MSH3*, *RPS20* and *MLH3* (Figure 1) [39,40,41,42], but still await independent validation or additional functional evidence. However, it is remarkable that through all NGS efforts, only few novel high-penetrant risk genes for hCRC and polyposis have been established in the past decade, such as *POLE*, *POLD1* and *NTHL1* [43,44]. A detailed description of the established and most promising candidate genes for hCRC and polyposis identified through NGS is reviewed elsewhere [45,46].

## 3. Strategies for Identification of Rare High-Penetrant Risk Genes

To discover rare but distinct monogenetic causes in a -now known as a- phenotypic heterogeneous group of hCRC and polyposis patients, robust discovery studies are needed. Up to August 2020, we retrospectively identified 37 whole-exome sequencing (WES) and whole-genome sequencing (WGS)-based studies that aimed to discover novel candidate risk genes for hCRC and/or polyposis (Table 1). We reviewed these studies following the general setup of candidate discovery studies, which cover cohort composition, variant discovery and prioritization, and variant validation (Table 1, Table S1). Six studies based discovery on the same cohort that was enlarged over time [53,64,69,72,75,76].

### 3.1. Discovery Cohort

For the discovery of high-penetrant risk genes for hCRC and polyposis, family history (FH) and inheritance patterns are key factors in variant discovery. We noted that among the 37 candidate gene discovery studies, FH-based inclusion criteria varied from study to study. Some studies used a relatively broad inclusion criterion such as “one first-degree relative or second-degree relative with CRC” while others applied more stringent criteria “the presence of at least three relatives with CRC, of which at least two in consecutive affected generations and at least one case diagnosed with CRC before the age of 60” (Table 1: Inclusion criteria FH) [42,43,48,50,53,56,59,64,69,70,72,75,76]. Furthermore, phenotypic characteristics strongly associated with hereditary CRC and polyposis syndromes, such as tumor types and age-of-onset strongly varied between, but also within cohorts (Table 1: Inclusion criteria index phenotype; Table 1: Inclusion criteria age). The phenotypes that were studied included either polyposis, familial colon and/or rectal cancer, or a mixture of the aforementioned phenotypes. Age-based inclusion criteria were applied in twelve out of the 32 unique discovery cohorts [13,43,47,49,53,54,56,59,64,65,67,69,70,71,72,75,76]. However, this age-based inclusion criterion was heterogeneous ranging from an age at diagnosis ≤35 years [47] to diagnosis <60 years [43], to at least one relative diagnosed <60 years [53,64,69,72,75,76]. The observed heterogeneity within these NGS study cohorts is in contrast with the discovery studies that were performed before the NGS-era, as discovery studies before the NGS-era were directed to families with multiple affected members and a strong phenotype of hCRC and/or polyposis. The elaborate inclusion of a range of phenotypes might have contributed to the limited number of high-penetrant risk genes discovered. Therefore, future candidate gene studies may benefit by composing clinical homogenous cohorts with respect to expected mode-of-inheritance and age-of-onset (Table 2).

### 3.2. Variant Prioritization

#### 3.2.1. Locus Prioritization

To identify novel genetic predispositions, WES or WGS data of discovery cohorts need to undergo a filtering to prioritize potentially pathogenic variants. In general, we observed prioritization based on either complete screening of WES or WGS data or, more targeted, by focusing on specific genes or regions that are more likely to be involved in hereditary cancer. The main applied variant prioritization strategies included linkage (*n* = 8 studies [41,43,52,55,61,66,73,75]), variants shared among affected relatives or absence of the variant in unaffected relatives (*n* = 19 studies [41,43,48,51,52,53,55,57,60,61,63,64,66,67,68,69,70,74,76]) and gene function (*n* = 14 studies [13,47,53,57,58,59,63,64,65,71,72,74,75,76]). Other approaches include the prioritization of recurrent variants (*n* = 7 studies [13,39,40,44,50,56,65]) and prioritization based on expected recessive or dominant mode-of-inheritance (*n* = 7 studies [52,53,54,60,62,67,71]). A minority of the studies primarily focused on deleteriousness of a variant (*n* = 6 studies [39,40,44,47,49,50]) or combined germline and tumor analysis (*n* = 1 studies [47]) (Table S1: Approach for discovery). Gene discovery studies that led to the identification of pathogenic variants in *POLE*, *POLD1*, and *NTHL1* were based on the prioritization of variants that were shared among affected family members or that were recurrent in the study population [43,44], suggesting that discovery cohort selection is most important for candidate gene discovery.

#### 3.2.2. Allele Frequency Cut-Offs

Additional to these aforementioned strategies, a commonly used approach among discovery studies is the “rare disease rare variant hypothesis”, meaning that rare phenotypes are caused by variants that are rare (i.e., have a (very) low MAF) in the general population. Within the 37 candidate gene discovery studies, the applied MAF cut-off ranged from 0.00–0.20 (Table S1: Applied MAF). Four studies explicitly adapted their MAF cut-off to presumed dominant or recessively inherited genetic predispositions in their study populations [40,44,62,71]. These four studies applied more stringent MAF cut-offs in dominant scenarios (MAF < 0.01–0.001) and looser MAF cut-offs for recessive inheritance patterns (MAF < 0.03–0.01) [40,44,62,71].

A prominent question in MAF-based filtering is ‘How low can we go?’. In other words, what is the optimal MAF frequency to identify rare potentially pathogenic variants for follow up? Assuming a high-penetrant rare disease model, in theory MAFs could be set as low as the expected prevalence of disease in the general population for dominant inheritance scenarios. The most recently discovered dominantly inherited polyposis syndrome proof-reading associate polyposis (PPAP), caused by variants in the exonuclease domain of *POLE* and *POLD1*, is discovered only in about 0.5% of the familial early-onset CRC disease population [77]. ClinVar Class V variants located in the exonuclease domains of *POLE* (codons 268-471; NM_006231) and *POLD1* (codons 304-517; NM_002691), such as *POLE* p.(Leu424Val) and *POLD1* p.(Ser478Asn) are absent in the general population [78]. Based on these observations, we argue that novel dominant hCRC and polyposis syndromes are likely just as rare in the general population as PPAP and therefore identification of novel dominant high-penetrant risk genes will allow (very) low variant allele frequency cut-offs. Chubb et al. screened a selected population of familial early-onset CRC cases, and concluded that about 0.5 percent of this population carries a pathogenic or likely pathogenic variant in *POLE* or *POLD1*. Based on the assumption that this population of familial early-onset CRC cases could completely be explained by high-penetrant predispositions and that up to 10% of the CRCs can be explained by rare genetic predispositions [8,77], we anticipate that novel high-penetrant variants for hCRC and polyposis will have a MAF lower than 0.0005, or will be completely absent in the general population.

For recessive disease scenarios, including compound heterozygous variants, setting a MAF cut-off is much more difficult as heterozygous variants can be expected in the general population in the absence of a disease phenotype. For recessive disease genes such as *MUTYH* and *NTHL1*, combined minor allele frequencies up to 0.007 (0.7%) are observed, and population-specific allele frequencies can be as high as 0.006 (North-Western European; *MUTYH* p.(Gly393Asp)) [44,79]. This observation implies that a MAF cut-off of 0.001, as often applied for dominant diseases, is too stringent. Based on these calculations, MAF cut-offs as low as 0.007 should be set for variants in recessive disease scenarios.

#### 3.2.3. In Silico Pathogenicity

Not every selected rare variant will have pathogenic –and thus a cancer predisposing– potential. Therefore, after rare variant selection, the pathogenicity potential of a variant needs assessment. Twenty-nine out of 37 studies reported the use of one or more in silico tools to predict variant pathogenicity [24,39,40,41,43,44,48,49,50,52,53,54,55,56,57,58,59,61,62,63,64,67,68,71,72,73,74,75,76]. In general, combinations of different variant scoring tools such as SIFT, PolyPhen-2, and MutationTaster were used (Table S1). Specific criteria for pathogenic assertion were applied in 21 out of 29 studies, such as prediction for deleteriousness in a majority of the assessed tools and application of cut-offs for CADD and/or PhyloP scores that indicate the conservation of a position [80,81,82]. The performance and concordance of such in silico tools for pathogenicity greatly varies [80,83]. In line with the observation that most studies make use of multiple in silico tools, a previous comparison of 25 commonly used algorithms showed that prediction of five algorithms (SIFT, PolyPhen, CADD, PROVEAN, and MutationTaster) resulted in a higher concordance compared to other combinations [80]. For example, known pathogenic missense variants such as *MUTYH* (NM_012222.2; c.724C > T; p.(Arg242His)) and *POLE* (NM_006231.2; c.1270C > G; p.(Leu424Val)) are predicted to be deleterious in all five algorithms. However, a high concordance of these in silico tools is not a guarantee for the identification of a pathogenic variant, therefore in silico predictions should facilitate variant prioritization but should not serve as evidence in itself.

#### 3.2.4. Co-Segregation

Next to germline-based filtering strategies and in silico pathogenicity predictions, additional prioritization methods are applied to select the most likely causative variant. One of the primary types of evidence is co-segregation of the variant with the affected status throughout a family. Co-segregation analysis was performed in 24 out of 37 studies (Table S1: Segregation [39,40,41,43,44,48,49,50,51,52,53,57,60,61,63,64,66,67,68,69,73,74,75,76]). Within the studies, co-segregation of the candidate risk locus was not always concordant in affected vs. unaffected relatives. Gylfe et al. identified the *TWSG1* nonsense variant (c.121C > T; p.(Gln41 *)) in two families, which segregated with the affected status in one family, but not in the other [50]. Jansen et al. showed that that all variants that were found in the affected individual (with CRC at age 14) were also detected in either the unaffected father or the unaffected mother of the proband [74]. Co-segregation analysis is considered essential for decisions in variant follow up, especially for discovery of novel high-penetrant risk genes. To illustrate, incomplete co-segregation of the *RAD52* truncating variant (c.590_593dupAACC; p.(Ser199Thrfs*88)), in contrast to the complete genotype-phenotype segregation of *FAF1* missense variant (c.1111G > A; p.(Asp371Asn)) in a family with CRC, led to the decision to follow up the latter one [76]. Co-segregation analysis of both affected and unaffected family members will rapidly gain insight for variant follow up. The lack of segregation in healthy family members can indicate variant pathogenicity as well, taking into consideration the age of the person and the expected age-of-onset of the disease. Even when co-segregation analysis in affected family members cannot be performed, testing of unaffected family members may facilitate variant prioritization.

### 3.3. Variant Validation

#### 3.3.1. Molecular Tumor Analysis

Somatic molecular events in colorectal tumors were studied in eleven discovery studies (Table S1: Molecular tumor characteristics [39,40,42,43,44,47,52,57,62,68,72]). Within these eleven studies, analysis of tumor mutational events varied from driver gene genotyping of *KRAS*, *BRAF*, and/or *NRAS* [39,42,68], to analysis of the genome-wide mutational spectrum of the tumor [43,44,57,72]. Driver gene analyses are often applied in the context of therapy-stratification and evolutionary studies of the tumor. However, driver genes were also screened in several candidate gene discovery studies as well. Nine out of these eleven studies performed driver gene screening, but the vast majority of these nine studies did not find any predominant substitution in the analyzed driver genes [39,43,44,47,52,68,84]. Gala et al. found enrichment for *BRAF* p.(Val600Glu) in sessile serrated adenomas [42]. *BRAF* p.(Val600Glu) mutations in tumors are strongly associated with *MLH1* promoter methylation in sporadic CRC cases and thus are a predictor of negative MMR mutation status [85]. Summarizing these study results, driver gene screening in hCRC and polyposis gene discovery studies may have additional value when screening is performed on tumors of all discovery cases to further stratify hCRC and polyposis phenotypes, as complete screening could facilitate the identification of sporadic cases and may provide an extra tumor characteristic that could stratify patients for follow up screening. Nonetheless, known driver gene mutations seem not to be discriminative for specific germline predispositions for polyposis, at least not in adenomatous polyposis.

Based on the established genes discovered for hCRC and polyposis, pathogenic variants in two main mechanisms can give rise to colorectal tumors. One is the altered activity of a tumorigenic process; i.e., inactivation of *APC* and activated WNT signaling, and the tumor suppressive roles of *SMAD4* and *BMPR1A* in TGF-β signaling. The other main mechanism that predisposes tumor development is a defect in DNA repair, such as MMR defects in Lynch syndrome, and disruptive base-excision repair (BER) in adenomatous polyposis. For DNA repair deficiencies in particular, it is known that germline defects in specific genes give rise to specific molecular tumor phenotypes. The most prominent molecular phenotype is the observation of microsatellite instability in the tumor due to (germline) MMR defects. Defects in DNA repair pathways may result in distinct mutational patterns in the genomes of tumors, now known as mutational signatures [86]. The observation of an increased rate of C:G > A:T transversions in *APC* in tumors led to the discovery of *MUTYH*-associated polyposis in the early 2000s. MUTYH deficiency, causing 8-OxoG BER pathway redundancy, is now linked to mutational signatures 18 and 36 [87,88]. Subsequently, in 2015, tumors of *NTHL1*-associated tumor syndrome (NATS) patients with germline nonsense mutations in the BER gene *NTHL1* showed an increased rate of C:G > T:A mutations in a unique mutation context, resulting in mutational signature 30 [89]. Polymerase proofreading defects give rise to mutational signatures 10a and 10b, and mismatch repair defects are associated with mutational signatures 6, 15, 20, and 26 [90,91]. These findings suggest that mutational patterns, rather than single driver gene events, may facilitate identification and validation of candidate genes for hCRC and polyposis syndromes (further reading: Grolleman et al., 2019 [92]).

#### 3.3.2. Functional Characterization of the Variant

Co-segregation of the variant and mutational profiling give a strong indication for pathogenicity, but these two aspects do not directly confirm the causality of the germline variant to the disease phenotype. Therefore, additional evidence may include the expression pattern of the affected gene and functional characterization of the variant. In this review, we consider functional characterization as the use of in vitro or in vivo assays to determine whether: (i) genetic variants disrupt or enhance protein function, but more importantly (ii) how an altered protein function may give rise to a certain phenotype. A combination of these two is likely essential for full functional characterization. Gene and/or protein expression alone cannot be considered as a validation method, as the presence of gene product does not determine the pathogenicity of the variant and the effect on down-stream targets. Overall, functional characterization of variants was limited in the reviewed candidate studies. In total, 17 out of 37 studies used patient-derived material or used human cell lines as an in vitro model to test variant consequences and functional characterization (Table S1: Candidate gene transcription/protein expression and Functional characterization [13,39,40,41,42,44,49,52,57,58,59,60,66,67,68,69,76]). However, most studies provide mainly protein expression data and limited data on assessment of protein function and downstream interactors [13,40,66,69], while both expression data as well as functional data should be in line with the role of the variant in tumorigenesis. For example, we previously showed that missense variants in *LRP6* did not affect expression and localization compared to wildtype *LRP6*. However, using a TOPflash assay we observed that these missense variants increased WNT-signaling activity [13]. In other studies, more in-depth functional analyses in tumorigenic processes included experiments to analyze the effect of variants on cell migration and proliferation, cell cycle progression, and apoptosis [52,68,76]. Schulz et al. showed increased activation of the PI3K/AKT and MAPK/ERK pathways for SEMA4A^V78M^ compared to SEMA4A^wt^ by flowcytometry and immunofluorescence in HCT116 cells, but not in 293T cells [52]. Bellido et al. showed a potential pathogenicity of *BRF1* missense variants as low viability was observed in a *BRF1*-dependent growth assay in yeast that harbored these missense variants [68]. Bonjoch et al. performed various assays including immunofluorescence and caspase-3 activity assay to show that *FAF1* missense variants lead to upregulation of β-catenin and reduced apoptosis in DLD1 cells [76]. In addition to two-dimensional models, colon epithelial organoid models may be an interesting alternative to study proliferation, survival and mutational processes for specific candidate predisposing genes in a three-dimensional setting. It was previously shown by Drost et al. that an organoid in which *NTHL1* was knocked out using CRISPR/Cas9 shows the same mutational pattern as tumors from NTHL1 deficient individuals [89,93]. Standardized methods have been developed for the in vitro culture of primary colon organoids, which may facilitate the use of this model over two-dimensional cultures [94]. Nevertheless, only a selected number of parameters can be studied with in vitro assays. Therefore, a combination of tests, including in vitro functional assays as well as tumor sequencing data and/or co-segregation analysis, should point towards a causal genotype-phenotype relationship (Table 2).

#### 3.3.3. Case-Control Validation

Next to functional impact of a candidate variant, case-control validation is an alternative and complementary approach to validate the causal relationship between a germline variant and the hereditary tumor syndrome. The validation is based on a significantly higher recurrence rate of the hCRC or polyposis syndrome phenotype in cases than controls. During the identification process of candidate genes, a low MAF in population controls is already used as selection parameter, however in case-control validations other variants in the same gene are also taken into consideration. Moreover, case-control studies contribute to the description of the complete phenotypic spectrum of a candidate gene. Validation cohorts have been used in 23 out of 37 studies, and statistical testing for enrichment in cases vs. controls was performed in fifteen studies [13,42,43,44,52,55,56,59,64,65,68,72,73,74,75]. Despite efforts, the lack of sufficient power is frequently mentioned as reason for not finding significant differences in case-control analysis and not being able to validate newly found risk genes. To illustrate, Chubb et al. performed a screening of 1,006 cases and healthy 1,609 individuals. Even though the cohort was specifically targeted to dominant hCRC syndromes by the selection of CRC cases ≤55 years and with at least one first-degree relative with CRC, only the well-established genes for hCRC and polyposis (*APC*, *MLH1*, *MSH2*) reached significant enrichment in cases versus controls [65]. In hCRC and polyposis syndromes, the rareness of the newly found syndromes together with their population-specific allele frequency as e.g., noted for *NTHL1* [79], show that the low MAFs of rare variants make it almost impossible to find significant associations, even in studies with over a thousand cases. It is calculated that for genetic predispositions with moderate or high-penetrance (OR > 2), required sample sizes need to reach 10,000 to even 100,000 cases and controls [65,95]. Therefore, the purpose of case-control validation in future studies may shift from finding significant differences in cases versus controls, to specification of the phenotype associated with the risk gene. Validation cohorts may include a phenotypic range of genetic tumor risk syndrome patients to further determine the genotype-phenotype presentation in these rare disease patients (Table 2).

## 4. Missing Heritability Explained by Known or Common Risk Genes

In the era of massive parallel sequencing, whole-exome and whole-genome sequencing of suspected hCRC and polyposis patients resulted in relatively few widely established novel high-penetrant genes for hCRC and polyposis syndromes. In the above sections, we gave insights into the applied methods and discussed considerations for future candidate gene discovery studies. However, next to the inability to identify novel genetic predispositions possibly due to inconsistencies in study setup, other scenarios could also contribute to the observed missing heritability in hCRC and polyposis syndromes. These scenarios include that disease-causing variants might have been missed in known hCRC and polyposis syndrome genes due to technical limitations or unforeseen inheritance patterns, or that the hCRC and polyposis phenotypes are the result of multiple variants in low- and moderate penetrant risk genes.

### 4.1. Identification of Variants in Known hCRC and Polyposis Risk Genes by Whole-Genome Sequencing

Most routine diagnostic, but also research facilities, focus on the screening of coding regions of the genome for pathogenic variant identification, either by targeted- or whole-exome sequencing. However, targeted screening of both the coding and non-coding regions of the genes or WGS could be of great potential for reduction of missing heritability. Short-read WGS outperforms WES in detection of variants also in coding regions due to more homogeneous coverage with higher quality and better variant calling [96,97]. Moreover, WGS of patient cohorts might facilitate discovery of missed non-coding variants in known hCRC and polyposis genes. In the past, deep-intronic and promoter variants were described in tumor suppressor genes *APC* and *PTEN*, which makes sequencing of these non-coding regions of particular interest for unresolved hCRC and polyposis patients [98,99,100,101,102,103]. Long-read sequencing and optical mapping techniques might be valuable as well, as these techniques are specifically directed to the detection of complex and structural variants, and allow alignment and variant mapping in regions that used to be uncovered in the past due to their nucleotide composition (e.g., extreme GC-rich, and multiple short repeats) [104,105]. Complexities of these regions and the structural variants itself, make that these variants remain understudied in whole exome-based and whole genome-based techniques, and the inability to detect those might explain part of the observed missing heritability in hCRC and polyposis syndromes.

### 4.2. Mosaic and De Novo Variant in Known hCRC and Polyposis Syndrome Genes

Next to dominant and recessive inheritance patterns, other forms of predisposition might also explain a proportion of the missing heritability in hCRC and polyposis patients. A de novo onset of a constitutive genetic defect and mosaicism, caused by mutations arisen in (early) embryonic development, are likely overseen causes in genetically unresolved hCRC and polyposis patients, as these patients often lack a positive family history. Despite a negative family history, these patients may display severe polyposis and carcinomas at young age. In example, de novo and mosaicism rates among FAP patients with suspected sporadic disease range from 4% to 25% [84,106,107,108,109]. For identification of novel genes involved in de novo onset of disease, trio-studies could be extremely valuable, as sequencing both healthy parents and the proband increases the diagnostic yield in rare-diseases [110]. Trio-sequencing may be chosen in case of a severe polyposis phenotype or CRC at an exceptionally young age, but in the absence of familial aggregation. For detection of mosaicism, multiple clonal expansions like polyps should be evaluated to determine shared pathogenic variants, which subsequently can be evaluated at high sensitivity in leukocyte-derived DNA and normal tissues to render insight in variant distribution throughout the different tissues in the body.

### 4.3. Polygenic Risk Scores

The influence of common, low-penetrant risk loci has been studied since the introduction of genome-wide studies. Genome-wide association studies use the genetic risk information from the millions of discovered single nucleotide polymorphisms to determine an individual’s genetic susceptibly for a specific, usually complex, trait. Using this information, the sum of all common, intermediate and rare variants that are thought to contribute to disease susceptibility. The interactions within and between these variants form a Polygenic risk score (PRS). PRSs have been studied in several complex traits as well as several cancers, including breast and prostate cancer [111,112]. Additionally for colorectal cancer, PRSs in combination with family history seem to be feasible for risk stratification [113]. However, little is known on the additional role of polygenicity in contribution to monogenic causes of hCRC and polyposis syndromes. A preliminary publication of Fahed et al. studied whether polygenic risk can account for variation among carriers for monogenic variants that are predisposed to Lynch syndrome and showed that the odds ratios for colorectal cancer increased with higher polygenic scores [114]. Research from Schlafly et al. shows a discovery approach using PRS to prioritize families for high-penetrant rare risk genes. Using this approach in 404 melanoma-prone families, they found that families carrying putative causal predisposition had a lower PRS [115]. It is too early for implementation, but both studies show the potential of PRSs as a tool to prioritize families for discovery cohort inclusion in hCRC and polyposis syndromes gene discovery studies.

## 5. Conclusions

A fair number of hCRC and polyposis patients are considered to remain genetically unexplained, which hampers risk assessments for patients in whom no genetically underlying cause is identified. The estimates of missing heritability are mainly based on twin and family studies, which may be biased by non-additive genetic effects or incorrect assumption about the shared environment. Nevertheless, the proportion of unresolved early-onset and/or familial CRC patients, urges the investigation of additional genetic causes. By collecting all whole exome-based and whole genome-based discovery studies and listing their study design, we aimed to provide knowledge on why the missing heritability is not (yet) reduced and provide improvements for future studies. These improvements cover the setup of high-quality discovery studies by including phenotypically well-defined early-onset CRC and/or polyposis syndrome patients into perhaps smaller, but more specific cohorts for candidate gene searches. In this approach, the availability of enough material and patient information should have the highest priority for inclusion to facilitate detailed characterization of both germline DNA and tumor material. Once a variant and/or candidate gene is selected, validation needs to be multi-leveled and elaborate to provide robust and unambiguous evidence for the casual role of the genetic variant.

In conclusion, novel high-penetrant risk genes for hCRC and polyposis syndromes will be rare in a disease group that is heterogeneous in nature. This heterogeneity needs to be taken into account in future discovery and validation strategies for the identification of novel genetic predispositions in hCRC and polyposis syndromes. A stringently selected study population and strict criteria for variant identification, together with appropriate functional validation, will contribute to a further delineation of the missing heritability. This complete analysis of this heterogeneous disease group will provide in-depth genotype-phenotype information, contributing to future diagnostics and lead to tumor- and patient-specific treatment and surveillance strategies.

## Figures and Tables

**Table 1:** Whole exome-based and genome-based rare high-penetrant risk gene discovery studies for hereditary colorectal cancer and polyposis syndromes up to August 2020.

Author	Key Gene(s)	Inclusion Criteria Index Phenotype	Inclusion Criteria Age	Inclusion Criteria FH	Size Discovery Cohort
Palles et al., 2013 [43]	*POLD1, POLE*	≥10 colorectal tumors	<60 years	FDR or SDR with CRC	15 families (20 cases)
Smith et al., 2013 [47]	*FANCM, LAMB4, PTCHD3, LAMC3, REX2*	Advanced CRC	≤35 years (18 cases)	No, sporadic	50 cases
DeRycke et al., 2013 [48]	*CENPE, KIF23*	Familial CRC	NS	≥2 members affected	16 families (40 cases)
de Voer et al., 2013 [49]	*BUB1, BUB3*	Non-polyposis MMR-proficient CRC	≤40 years	NS	33 cases
Gylfe et al., 2013 [50]	*UACA, SFXN4, TWSG1, PSPH, NUDT7, ZNF490, PRSS37, CCDC18, PRADC1, MRPL3, ARK1C4*	Familial CRC	NS	≥1 FDR with CRC	96 cases
Gala et al., 2014 [42]	*RNF43*	Sessile serrated adenomas	NS	If <5 SSAs, ≥1 FDR with SSAs or CRC	20 cases
Rohlin et al., 2014 [51]	No novel genes found, *POLE* found	Hereditary CRC	n/a	n/a	1 family (3 affected, 1 unaffected)
Nieminen et al., 2014 [41]	*RPS20*	Amsterdam/Bethesda FCCTX	n/a	n/a	1 family (4 cases)
Schulz et al., 2014 [52]	*SEMA4A*	Amsterdam I FCCTX	n/a	n/a	1 family (4 cases)
Esteban-Jurado et al., 2015 [53]	*CDKN1B, XRCC4, EPHX1, NFKBIZ, SMARCA4, BARD1*	Familial CRC ^1^	≥1 relative diagnosed <60 ^1^	≥3 affected, ≥2 in consecutive generations	29 families (43 cases) ^1^
Tanskanen et al., 2015 [54]	*ADAMTS4, CYTL1, SYNE1, MCTP2, ARHGAP12, ATM, DONSON, ROS1*	Non-syndromic early-onset CRC	<40 years	NS	22 cases
Wei et al., 2015 [55]	*HNRNPA0* and *WIF1*	Multiple early-onset cancer	n/a	n/a	1 family (4 affected, 4 unaffected)
Zhang et al., 2015 [56]	*EIF2AK4, LRP5, BUB1*	Familial CRC without polyposis	≤55 years	If ≥40 years, ≥1 FDR with CRC	21 families (23 cases)
Weren et al., 2015 [44]	*NTHL1*	Multiple adenomatous polyps	NS	NS	51 cases (48 families)
Segui et al., 2015 [57]	*FAN1*	Amsterdam I MMR-proficient CRC	n/a	n/a	1 family (3 cases)
Ngeow et al., 2015 [58]	*SMAD9*	HPS	n/a	n/a	1 family (1 case)
Arora et al., 2015 [59]	*ERCC6, WRN*	CRC or polyposis (≥10 polyps)	<50 years	≥1 relative with CRC	25 cases
Goldberg et al., 2015 [60]	*MCM9*	Multiple mixed polyposis and metastatic CRC	n/a	n/a	1 family (1 cases, 1 unaffected)
Rohlin et al., 2016 [61]	No novel genes found, *GREM1* and *POLE* found	AFAP/atypical polyposis	n/a	n/a	1 family (4 affected, 4 unaffected cases)
Spier et al., 2016 [62]	*DSC2, PIEZO1*	Colorectal adenomatous polyposis	NS	NS	7 cases
Thutkawkorapin et al., 2016 [63]	*DZIP1L, IGSF10, NOTCH1, SF3A1, GAL3ST1*	Familial rectal- and gastric cancer	n/a	n/a	1 family (3 cases)
de Voer et al., 2016 [13]	*PTPN12, LRP6*	non-polyposis MMR-proficient CRC	≤45 years	NS	55 cases
Esteban-Jurado et al., 2016 [64]	*BRCA2/FANCD1, BRIP1/FANCJ, FANCC, FANCE, REV3L/POLZ*	Familial CRC ^1^	≥1 relative diagnosed <60 ^1^	≥3 affected, ≥2 in consecutive generations	40 families (74 cases) ^1^
Chubb et al., 2016 [65]	*POT1, POLE2, MRE11*	CRC	≤55 years	≥1 FDR with CRC	1006 cases
Adam et al., 2016 [40]	*MSH3*	≥20 synchronous or ≥40 metachronous colorectal adenomas	NS	NS	102 cases
Schubert et al., 2017, 2018 [66]	*MIA3*	Amsterdam I MMR stable familial CRC	n/a	n/a	1 family (3 cases WES, 2 cases WES/WGS, 1 cases WGS)
Martín-Morales et al., 2017 [67]	*SETD6*	Amsterdam I FCCTX	≥1 relative diagnosed <50	≥3 affected (≥1 FDR), ≥2 in consecutive generations	1 family (2 cases, 1 unaffected)
Bellido et al., 2018 [68]	*BRF1*	Amsterdam I hereditary CRC	n/a	n/a	1 family (3 CRC cases, 1 BC case)
Franch-Expósito et al., 2018 [69]	*TTF2, TMEM158*	Familial CRC ^1^	≥1 relative diagnosed <60^1^	≥3 affected, ≥2 in consecutive generations	WES: 38 families (71 cases), WGS: 1 case ^1^
Yu et al., 2018 [70]	*DDX20, ZFYVE26, PIK3R3, SLC26A8, ZEB2, TP53INP1, SLC11A1, LRBA, CEBPZ, ETAA1, SEMA3G, IFRD2* and *FAT1*	Amsterdam I/II non-polyposis hereditary CRC	≥1 relative diagnosed <50	≥1 FDR & 2 generations affected	1 family (3 cases)
Olkinuora et al., 2018 [39]	*MLH3*	Adenomatous polyposis	NS	NS	40 cases
Thutkawkorapin et al., 2019 [71]	*BMPR1A, BRIP1, SRC, CLSPN, SEC24B, SSH2, ACACA, NR2C2, INPP4A, DIDO1, ATP10B, PKHD1, UGGT2, MYH13, TFF3*	Simplex early-onset CRC	<40 years	NS	51 cases
Diaz-Gay et al., 2019 [72]	*BRCA2, BLM, ERCC2, RECQL(=WRN), REV3L* and *RIF1*	Familial CRC ^1^	≥1 relative diagnosed <60 ^1^	≥3 affected, ≥2 in consecutive generations	18 cases ^1^
Toma et al., 2019 [73]	*FBLN2*	Familial CRC/SPS	NS	≥2 affected in consecutive generations	16 families (39 cases)
Jansen et al., 2020 [74]	*NOTCH2, RAB25*	Familial CRC	NS	NS	5 families (9 cases)
Toma et al., 2020 [75]	*SMO*	Familial CRC ^1^	≥1 relative diagnosed <60 ^1^	≥3 affected, ≥2 in consecutive generations	18 families (47 cases) ^1^
Bonjoch et al., 2020 [76]	*FAF1*	Familial CRC ^1^	≥1 relative diagnosed <60 ^1^	≥3 affected, ≥2 in consecutive generations	40 families (75 cases) ^1^

Abbreviations: AFAP = Attenuated familial adenomatous polyposis, BC = Breast cancer, CRC = colorectal cancer, FCCTX = familial colorectal cancer type X, FDR = first degree relative, FH = family history, HPS = hamartomatous polyposis, MMR = mismatch repair, NS = not stated in article, n/a = not applicable, SDR = second degree relative, SPS = serrated polyposis syndrome, SSA = sessile serrated adenoma, WES = Whole-exome sequencing, WGS = Whole-genome sequencing. ^1^ Overlapping cohorts.

**Table 2:** Summary of considerations for future candidate gene discovery studies.

**Discovery Cohort Selection**
Clinical homogeneous cohorts based on the expected mode-of-inheritance and/or age-of-onset
**Variant Prioritization**
Locus prioritization based on variant recurrence within the cohort
Set allele-frequency cut-offs based on expected mode-of-inheritance - Dominant: MAF < 0.0005; Recessive MAF < 0.007
Concordance of multiple in silico prediction tools
Co-segregation among family members
**Variant Validation**
Molecular tumor analysis to determine molecular phenotype of tumor
Evidence based on cellular models
Case-control comparison to specify the genotype-phenotype correlation

MAF = Minor Allele Frequency.

## Supplementary Materials

The following are available online at https://www.mdpi.com/1422-0067/21/22/8757/s1. Table S1: Variant discovery and validation characteristics of whole genome and whole exome based candidate gene discovery studies for hCRC and polyposis.

## Abbreviations

AFAP	Attenuated familial adenomatous polyposis syndrome
BER	Base-excision repair pathway
CRC	Colorectal cancer
FAP	Familial adenomatous polyposis
FH	Family history
hCRC	Hereditary colorectal cancer
MAF	Minor allele frequency
MAP	MutyH-associated polyposis
MMR	Mismatch repair
NATS	*NTHL1*-associated tumor syndrome
NGS	Next-generation sequencing
PPAP	Polymerase proofreading associated polyposis
PRS	Polygenic risk score
WES	Whole exome sequencing
WGS	Whole genome sequencing
